# RbfA Is Involved in Two Important Stages of 30S Subunit Assembly: Formation of the Central Pseudoknot and Docking of Helix 44 to the Decoding Center

**DOI:** 10.3390/ijms22116140

**Published:** 2021-06-07

**Authors:** Elena M. Maksimova, Alexey P. Korepanov, Olesya V. Kravchenko, Timur N. Baymukhametov, Alexander G. Myasnikov, Konstantin S. Vassilenko, Zhanna A. Afonina, Elena A. Stolboushkina

**Affiliations:** 1 Institute of Protein Research, Russian Academy of Sciences, 142290 Pushchino, Russia; elena.maks.89@gmail.com (E.M.M.); korepan@vega.protres.ru (A.P.K.); olesyak@vega.protres.ru (O.V.K.); ori224@mail.ru (Z.A.A.); 2 National Research Center, “Kurchatov Institute”, Akademika Kurchatova pl. 1, 123182 Moscow, Russia; baymukhametov.timur@gmail.com; 3 Petersburg Nuclear Physics Institute named by B.P. Konstantinov of NRC “Kurchatov Institute”, 188300 Gatchina, Russia; alexander.myasnikov@stjude.org; 4 Department of Structural Biology, St. Jude Children’s Research Hospital, Memphis, TN 38105, USA

**Keywords:** ribosome assembly, cryo-EM, 30S subunit maturation, RbfA, two-dimensional gel electrophoresis

## Abstract

Ribosome biogenesis is a highly coordinated and complex process that requires numerous assembly factors that ensure prompt and flawless maturation of ribosomal subunits. Despite the increasing amount of data collected, the exact role of most assembly factors and mechanistic details of their operation remain unclear, mainly due to the shortage of high-resolution structural information. Here, using cryo-electron microscopy, we characterized 30S ribosomal particles isolated from an *Escherichia* *coli* strain with a deleted gene for the RbfA factor. The cryo-EM maps for pre-30S subunits were divided into six classes corresponding to consecutive assembly intermediates: from the particles with a completely unresolved head domain and unfolded central pseudoknot to almost mature 30S subunits with well-resolved body, platform, and head domains and partially distorted helix 44. The structures of two predominant 30S intermediates belonging to most populated classes obtained at 2.7 Å resolutions indicate that RbfA acts at two distinctive 30S assembly stages: early formation of the central pseudoknot including folding of the head, and positioning of helix 44 in the decoding center at a later stage. Additionally, it was shown that the formation of the central pseudoknot may promote stabilization of the head domain, likely through the RbfA-dependent maturation of the neck helix 28. An update to the model of factor-dependent 30S maturation is proposed, suggesting that RfbA is involved in most of the subunit assembly process.

## 1. Introduction

Ribosome biogenesis is a complicated multi-stage process comprising synthesis and maturation of ribosomal RNA (rRNA), the synthesis, modification, and folding of ribosomal proteins (r-proteins), and consecutive assembly of ribosomal subunits. Ribosome biogenesis is facilitated by numerous protein assembly factors: GTPases, RNA helicases, molecular chaperones, rRNA modification enzymes, etc. These factors help to avoid kinetic traps during RNA folding [1], assist the attachment of ribosomal proteins and prevent their premature and non-native binding [2]. However, the exact role of many biogenesis factors is still unclear and is to be determined.

The focus of this article is to study the well-known ribosome assembly factor ribosome-binding factor A (RbfA). RbfA has been identified as a bacterial cold shock response protein required for the efficient processing of the 16S rRNA 5′ end during the assembly of the 30S ribosomal subunit. This small single-domain protein (13–15 kDa) is found in most archaebacteria and eubacteria [3,4]. RbfA binds to 30S, but not to 50S ribosomal subunit or the 70S monosome [5,6]. The *rbfA* gene was originally isolated as a multi-copy suppressor for the cold sensitivity of a C23U mutation at the 5′ terminal helix of 16S rRNA [7]. RbfA is expressed constitutively under normal growth conditions; however, the expression level rapidly increases upon cold shock [6,8]. The elevated level of RbfA under cold-shock conditions is necessary to overcome the translational block at reduced temperature, presumably by promoting rapid maturation of 30S subunits [9]. A strain with *rbfA* deletion reveals slower bacterial cell growth and accumulates free ribosomal subunits [5] and 17S rRNA–16S rRNA precursors [10].

Three-dimensional structures of RbfA from different bacteria have been determined by X-ray [9; PDB ID 1JOS] and by NMR in solution [3,4; PDB ID 7AFQ; PDB ID 2KZF]. RbfA has type-II KH-domain fold topology, characteristic of the nucleic acid-binding protein family [11]. This domain consists of three α helices (α1–α3) and three β strands (β1–β3) with αββααβ topology [3]. In RbfA, α2 and α3 are arranged to form a helix–kink–helix (hkh) where the GxxG motif is replaced by an AxG sequence with a strongly conserved Ala residue forming an interhelical kink. In the RbfA complex with a mature 30S subunit, the hkh motif, encompassing α2 and α3, faces the junction between 16S rRNA helices (h) 44 and 45 [9]. A low-resolution cryo-electron microscopy (cryo-EM) structure of the 30S•RbfA complex showed that RbfA binds in the neck region, being buried deeply within the cleft between the head and the body of the 30S subunit [9]. The binding of RbfA induces a shift of h44 and h45 apexes, making the 30S•RbfA complex unsuitable for binding the 50S subunit. This complex is considered to be the most accurate representation of the post-processing state of the 30S subunit, just before RbfA dissociation. It seems likely that the bound RbfA prevents precursor 30S subunits with an unformed h1 helix and still unprocessed 5′ end from entering the translation initiation cycle.

Using X-ray-induced hydroxyl radical footprinting, it was shown that nucleotides in helices 1 and 2 of the central pseudoknot (PK) and in helix 44 of the decoding center became exposed in 30S intermediates accumulated in Δ*rbfA* cells [12]. Affinity purification of 17S rRNA containing Δ*rbfA* pre-30S subunits revealed the presence of two types of particles separable by sucrose gradient centrifugation, named “light” and “heavy” intermediates [12]. The “light” pre-30S subunits were unable to catalyze the formation of a peptide bond while the “heavy” ones produced dipeptides. However, structural differences in these Δ*rbfA* pre-30S complexes were not defined. Cryo-EM reconstruction of 30S intermediates from double mutant Δ*rbfA*Δ*rsgA* cells revealed the flexible 3′ head and 3′ minor domains including h44 and h45 [13]. However, the contribution of *rbfA* deletion to the structural distortion to 30S particles in this double mutant remained unclear.

Here, we report cryo-EM structures of 30S assembly intermediates accumulated in an *rbfA* null *Escherichia coli* strain. Deletion of the assembly factor RbfA caused a substantial distortion of an important central pseudoknot structure. It was shown that the relative order of the assembly of the 3′ head domain and the docking of the functionally important helix 44 depends on the presence of RbfA. The cryo-EM maps for pre-30S subunits were divided into classes corresponding to consecutive assembly intermediates: from the particles with a completely unresolved head domain and unfolded central pseudoknot to almost mature 30S subunits with partially distorted helix 44. Cryo-EM analysis of Δ*rbfA* 30S particles revealing the accumulation of two predominant classes of early and late intermediates allowed us to suggest that RbfA participates in two stages of 30S subunit assembly and is more deeply involved in the maturation process than previously thought.

## 2. Results

### 2.1. Characterization of the rbfA Null Strain of E. coli and Protein Complement of Pre-30S Particles from *∆*rbfA Cells

For the isolation of immature 30S particles, we used the *rbfA* null strain from the Keio collection [14], a library of single-gene deletions of nonessential genes in the *E. coli* K12 strain. The ∆*rbfA* strain displayed a cold sensitivity phenotype (slow bacterial growth at low temperatures), an altered ribosome profile (increased pool of free ribosomal subunits due to dissociation of 70S ribosomes), and a prevalence of immature 17S rRNA, as described previously (Supplementary Materials Figure S1) [5,10]. 30S ribosomal subunits from wild-type (wt) and ∆*rbfA E. coli* strains were purified using a published procedure with minor modifications [15].

**Figure 1:**
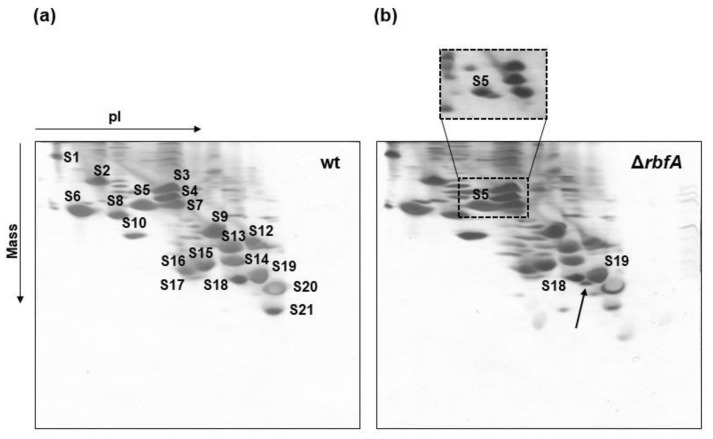
Immature 30S particles of Δ*rbfA* strain contain all the r-proteins present in 30S subunits purified from wild-type *E. coli* cells. Two-dimensional electrophoretic analysis of the protein composition of 30S subunits from wild-type strain (**a**), and from a strain with deleted *rbfA* gene (**b**). Proteins were separated by pI and by mass in 2D urea gel. The arrow indicates the position of non-acetylated S18. The inset represents the part of a similar gel with less protein loaded which demonstrates splitting of the S5 spot due to incomplete acetylation (**b**).

We used two-dimensional gel electrophoresis (2D electrophoresis) to cross-compare the r-protein composition of pre-30S particles purified from the ∆*rbfA* strain with those of a wild-type 30S preparation (Figure 1). All ribosomal proteins present in wild-type 30S subunits were identified in the immature Δ*rbfA* 30S particles. The only significant differences on the electrophoregram of the pre-30S subunits were additional signals running slightly faster in both dimensions than the spots attributed to proteins S5 and S18. Mass spectrometry analysis proved that these were the derivatives of proteins S5 and S18 (data not shown) and their increased mobility in the minus pole direction in the urea PAGE should be ascribed to the increased overall positive charge. Previously, it was found [16,17] that the N-terminal residues of the *E. coli* S5 and S18 proteins are acetylated, and the absence of these acetyl group would increase the positive charge without significant change in the molecular mass. It was also shown that S5 and S18 mutants lacking the N-terminal acetyl group do indeed run slightly faster on 2D electrophoregrams than their modified counterparts [18,19,20]. Here, a similar splitting of the S5 and S18 spots was observed under similar electrophoretic conditions, indicating the coexistence of acetylated and non-acetylated forms of these proteins.

The data that S5 and S18 were not fully acetylated in Δ*rbfA* pre-30S particles were obtained previously by MALDI-TOFMS [12] and it was suggested that the extent of S18 and S5 modification correlates with the formation of specific rRNA interactions during 30S assembly. It has been shown that in 30S subunits with a disrupted pseudoknot protein, S5 is undermodified [20]. Although the exact function of S18 acetylation has not yet been elucidated, it is known that S18 binds helix 22 and helps to stabilize the platform of the 30S subunit [21]. Our results indicate that 30S intermediates from RbfA-lacking cells probably have unformed structural elements in the pseudoknot and platform.

### 2.2. RbfA Participates in the Formation of the Central Pseudoknot

**Figure 2:**
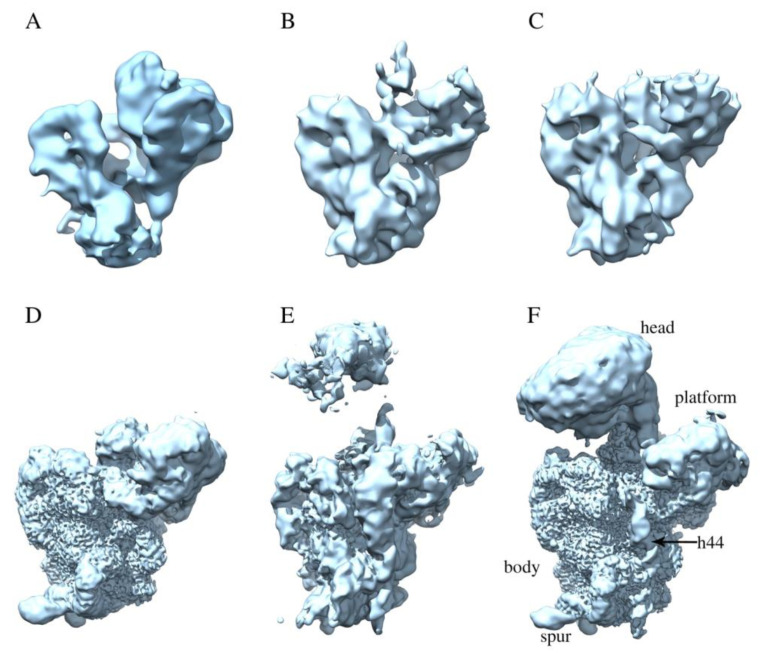
The sequence of intermediates on the pathway for the 30S subunit assembly. Cryo-EM maps for six classes of immature 30S particles accumulating in the Δ*rbfA* strain (**A**–**F**), representing 8, 5, 5, 30, 12, and 39% of the particle population, respectively. The average resolution of electron density maps obtained for classes (**A**–**C**) and (**E**) was limited to 8 Å. The cryo-EM structures for the two most common classes (**D**,**F**) were determined at a 2.7 Å resolution.

The structures of the immature 30S subunits purified from Δ*rbfA* cells were obtained using single particle cryo-EM, image classification, and three-dimensional (3D) reconstruction techniques. Multi-round 2D classification revealed very slight (<3%) contamination of the preparation with 50S subunits. The dataset was subjected to multiple rounds of 3D image classification, and immature 30S subunits were sorted into six well-defined classes, namely A, B, C, D, E, and F, covering 99% of the particle population and the electron density maps for each of these classes were obtained (Figure 2). None of the six classes represented the mature 30S ribosomal subunit. We succeeded in determining the cryo-EM structures for the two most common classes, D and F (total 69% of particle population), at a high resolution (2.7 Å). In addition, 3D cryo-EM reconstruction of the structure of mature 30S subunits purified from wild-type *E. coli* cells was performed and the obtained data were used as a reference to evaluate structural defects in the immature particles.

**Figure 3:**
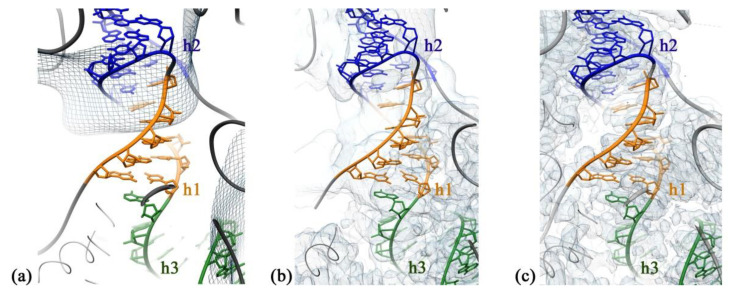
Сentral pseudoknot region in the cryo-EM structure of class A, D, and F intermediates. (**a**) The superposition of the atomic model built for the reference wild-type 30S subunit and сryo-EM density map for class A. The densities for helices 1 and 3 are completely missing and helix 2 is smeared. (**b**) Atomic model and сryo-EM density map for class D. Helices h1, h2, h3 are color-coded. (**c**) Atomic model and сryo-EM density map for class F.

Classes A, B, C consisted of immature Δ*rbfA* 30S subunits of a similar structure, representing 8%, 5%, and 5% of the particle population, respectively. Although the average resolution of electron density maps obtained for each of these classes was limited to 8 Å, they provided sufficient detail to visualize the general fold of 16S rRNA domains. All three classes represent intermediates with well-defined body density, a more poorly resolved platform, and a complete lack of the head domain that is, apparently, just starting to fold. Analysis of cryo-EM density maps of A, B, and C class averages revealed missing helices 1, 3, 27, and 44 in the decoding center, smeared densities for h2, h45, “neck” helix 28, and helices 22 and 23 that anchor the base of the platform (Figure 3), fragmented densities for r-proteins S4, S5 in the body and S6, S11 in the platform, and the complete absence of S21 and S1. The most notable is the absence of helix 44, which is essential for the formation of the decoding center, and of the central pseudoknot, consisting of helices 1 (nts 9–13/21–25) and 2 (nts 17–19/916–918), that connects three major domains of 16S rRNA and is necessary for ribosome stability and essential for the formation of the 30S initiation complex [20]. We interpreted the absence of these 16S elements in the density maps as an indication of an intermediate state with the central pseudoknot still unformed. Fragmentary structural data obtained for r-proteins are likely due to their intrinsic flexibility caused by maturation events still ongoing in the body and the platform.

The structure of immature Δ*rbfA* 30S subunits belonging to the second most common class D (30%) was resolved at a 2.7 Å resolution. Here, the densities corresponding to the head domain and helix 44 were also completely missing in the cryo-EM map of this class. However, in this case, the entire rRNA density was present in the body and the platform. Local resolution analysis showed that in the particles of class D, the body is the most defined region, whereas the platform is less well resolved. The densities for the proteins S11 in the platform and S5 in the body were fragmented and the densities for S21 in the platform and S1 were completely missing. These results indicate that in this intermediate, some parts of the body and the platform are still not completely formed while helices 1 and 2 have already acquired mature conformation. The acquired data suggest that the intermediates belonging to A, B, and C classes could precede intermediates of class D in the course of 30S subunit assembly.

### 2.3. RbfA Is Necessary for Correct Docking of Helix 44

**Figure 4:**
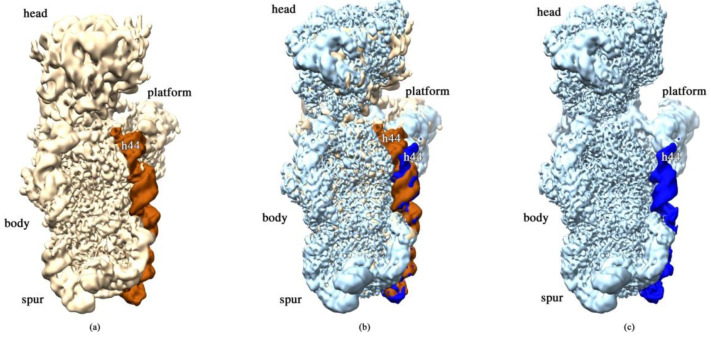
RbfA is responsible for the correct docking of 16S RNA helix 44. Cryo-EM maps obtained for (**a**) wt *E. coli* 30S subunit (h44 helix colored in orange) and (**c**) *E. coli* Δ*rbfA* pre-30S subunit (h44 helix colored in dark blue); (**b**) superposition of the maps (**a**,**b**).

Particles of classes E (12%) and F (39%) conform to more mature assembly intermediates with well-resolved body, platform, and head domains. Cryo-EM maps for these classes are similar to each other, and the main differences are in the resolution and position of the head domain. The map for class E has smeared densities at the head domain location, and only strongly fragmented densities for S9, S10, S14, and S19 proteins are visible while the densities for S2, S3, S7, and S13 are missing. Moreover, the head in the map for class E tilts backward relative to the body compared to class F. The head domain is well resolved in the map for class F, and only the densities for proteins S3, S7, and S13 are weak and the density for S10 is fragmented. In the platform, the density for protein S11 is also fragmented and the densities for S1 and S21 proteins are completely absent in both classes. In addition to the appearance of the head domain, the density for helix 44 of the 3′ minor domain (nts 1407–1494) is ordered and well-resolved in the maps for these two classes, although the map lacks the clear density for the flanking 1398–1406 and 1495–1498 16S RNA regions. However, the reconstruction revealed significant distortion of the overall structure of the 3′ minor domain, including helix 44. In particular, the upper segment of helix 44, which is responsible for tRNA binding, is displaced outward and protrudes from the interface side of the 30S subunit that is in contact with the 50S subunit (Figure 4). In the mature 30S subunit, this structural motif forms a part of the decoding center and bends toward helix 45 and the 3′ terminus of the 16S rRNA. In the intermediates of classes E and F, the displacement of helix 44 also leads to the shift of helix 45. These structural changes indicate that the decoding center is still not completely formed. Moreover, in the map for class F, the head of the pre-30S intermediate tilts backward by 10° compared to the wild-type 30S subunit.

Despite the lack of densities for proteins S1 and S21 in the maps for all the classes, the 2D electrophoretic analysis revealed the presence of all ribosomal proteins in the preparation of immature Δ*rbfA* 30S subunits. Based on an analysis of Δ*rbfA* pre-30S structures and 2D electrophoresis data (Figure 1), we concluded that most of the r-proteins flexibly interact with immature subunits and cannot be completely resolved by cryo-EM analysis. At the same time, one cannot exclude the possibility that S1 and S21 proteins may be absent in pre-30S particles prepared for cryo-EM analysis. It was previously shown that the exposure of 30S subunits to the air–water interface in the cryo-EM grid caused the loss of r-protein S2. The additional layer of continuous carbon on the grids removed this effect and the differences in 16S rRNA conformation were negligible [22]. This means that under certain conditions, cryo-EM sample preparation itself can cause the dissociation of the most labile ribosomal proteins, which, however, does not lead to significant changes in 16S rRNA structure. S1 and S21 are considered to be the late proteins of ribosome assembly, interacting rather weakly with the immature 30S subunit. It is likely that preparation of the pre-30S cryo-EM samples can cause the loss of these r-proteins. This is in agreement with cryo-EM data on the structures of the immature 30S subunits from other assembly factor knock-out strains (Δ*rimM*, Δ*yjeQ*, Δ*era*, ∆*rsgA*∆*rbfA*), where densities for S1 and S21 proteins are also absent [23,24,25,26].

Density for protein S2 is clearly determined only in the map for Δ*rbfA* pre-30S particles of class F. This is in good agreement with previous cryo-EM studies on the immature 30S Δ*yjeQ* subunits [23] where the map for the structure with helix 44 close to its native position showed S2 density similar to that in the mature structure. These data are consistent with the interpretation we propose that the most developed 30S particles of class F represent an immature pre-30S subunit that is rather close to the mature state.

Previously, it was shown that in immature 30S subunits purified from Δ*rbfA* cells, both ends of 17S rRNA remain unprocessed [24]. However, similar to previous cryo-EM studies on the immature 30S subunits [23,24,25], we failed to detect in cryo-EM maps of our dataset significant densities that could be attributed to extra nucleotides at 5′ and 3′ rRNA ends, which indicates structural instability of the 16S rRNA precursor terminal sequences.

We concluded that in the absence of RbfA, the immature 30S subunits have a very flexible head and distorted helix 44 with displaced helix 45. The head is connected to the body by the “neck” helix 28 of 16S rRNA which forms a continuous coaxial stack with helices 1, 2, and 3 of the body domain [21]. Unresolved head domains in the classes A, B, C, and D and missing and smeared densities for helices 1, 2, and 3 in the classes A, B, and C indicate that in the pre-30S subunits of the Δ*rbfA* strain, formation of the central pseudoknot and folding of the head are hampered. Therefore, RbfA may have a key role in the stabilizing of both the pseudoknot and the head. In addition, helices 44 and 45 are not completely formed in all the pre-30S classes, suggesting that their maturation also depends on RbfA. The role of RbfA could be the prevention of helix 44 docking against the body before the formation of the central pseudoknot. The fact that the most of Δ*rbfA* 30S intermediates were divided between two major classes—with and without a head and h44 helix (39 and 30% of the particle population, respectively)—is consistent with the major role of RbfA in several stages of 30S assembly: early formation of the central pseudoknot including folding of the head, and positioning of h44 in the decoding center at a later stage.

## 3. Discussion

The combination of genetic modification with cryo-EM analysis is widely used to identify the role of protein factors in assisting specific steps of the ribosome assembly process. The approach consists of creating a strain with knockout or depletion of a single assembly factor gene to disable or slow down the ribosome biogenesis process. Such cells accumulate immature ribosomal particles whose structural characterization by cryo-EM reveals the information about the reactions catalyzed by the corresponding factors. The main conclusion that was made based on this type of experiment was that most of the assembly factors assist in the maturation of the functional core of the 30S subunit—the decoding center [27]—located at the upper part of the body and the lower part of the head. It includes the upper part of h44, h45, the switch helix 27, the neck helix 28, and the central pseudoknot, consisting of helices 1 and 2.

Here, we isolated the immature 30S subunits from an *E. coli* strain lacking the *rbfA* gene and characterized them by cryo-EM. Six classes of the Δ*rbfA* 30S intermediates were defined that could be interpreted as milestones of the assembly pathway, starting from body formation with unfolded decoding center and poorly resolved platform and developing stepwise to an almost mature 30S subunit with just partially distorted helix 44. This interpretation does not contradict the generally accepted assumption that the 5′ body domain is formed first, followed by the central platform and head domains, and, finally, by the 3′ minor domain with the functionally important helix 44 in the decoding center [27]. Previously, the 30S assembly intermediates lacking the head domain have been found in the Δ*rbfA* cells and named Group I but have not been studied in detail [28]. We demonstrated two types of pre-30S intermediates with a completely unresolved head domain. The first type of the Δ*rbfA* 30S intermediates represents 18% of the particle population (classes A, B, C, similar to each other) and have an unfolded decoding center including disordered central PK, with completely missing densities for helices 1, 3, 27, and 44 and smeared densities for helices 2, 28, and 45. Additionally, the platform is not completely formed, since the cryo-EM maps of A, B, and C class averages revealed smeared densities for helices 22 and 23. These cryo-EM data are consistent with 2D electrophoretic analysis of Δ*rbfA* 30S particles, indicating underacetylation of proteins S5 and S18, which stabilize the pseudoknot and the platform, respectively. In summary, our results indicate that the absence of the assembly factor RbfA destabilizes the central pseudoknot, which is consistent with in vivo 16S rRNA hydroxyl radical footprinting experiments that revealed strongly exposed nucleotides in helices 1 and 2 in Δ*rbfA* cells [12]. A solution of the cryo-EM structure of the mature 30S subunit complex with RbfA revealed that the C-terminus of RbfA approaches 16S rRNA helix 1 [9]. In addition, in the precursor of 16 rRNA, helix 1, competes with an alternative secondary structure formed by leader sequences [7], which also reduces the stability of the central PK. Apparently, RbfA is involved in the re-structuring of the 5′ leader sequence of pre-16S rRNA, which allows the central PK to be formed.

The second type of Δ*rbfA* 30S intermediate with missing density for the head belongs to the second most populated class D (30%) and represents the particles consisting of the platform and the body with well-resolved PK and helices 3 and 27, and missing h44 and h45. The structure of immature Δ*rbfA* 30S subunits belonging to class D was resolved at a 2.7 Å resolution. The absence of the head domain, which, apparently, was just starting to fold, and the presence of structured central PK in class D intermediates indicate that the pseudoknot formation occurs before the start of the head domain folding.

Our data suggest that the deletion of *rbfA*, leading to the instability of central PK, hampers folding of the head domain. Analysis of the structure of the 30S•RbfA complex [9] showed that RbfA binds to the neck region of the 30S subunit, i.e., to a junction point between all three domains of the 30S subunit. Based on the position of RbfA in the 30S•RbfA complex [9] and the impeded folding of the head domain observed in this study, we suggest that RbfA promotes the maturation of neck helix 28 in the decoding center. Indeed, the smeared densities for helix 28 were detected only in earlier intermediates of classes A, B, and C with unformed PK; the fragmented density for this helix appeared in class D with folded PK and a full-size neck helix was found in late intermediates of classes E (12%) and F (39%) in which the head is anchored to the body and the platform. Cryo-EM maps for E and F classes are generally similar to each other, but they differ in the position of the head domain and the value of resolution obtained (8 Å and 2.7 Å, respectively). Intermediates of class E have an extremely flexible head domain that tilts backward relative to the body compared to those of class F particles. The most populated class F represents a pre-30S subunit that is rather close to the mature state. The densities for helices 44 and 45 of the 3′ minor domain also appear in the cryo-EM maps for classes E and F. The lack of RbfA alters the position and conformation of helix 44 dramatically and leads to the shift of helix 45 (Figure 4). Helix 44 is a functionally important segment of the 16S rRNA that is directly involved in mRNA decoding and the formation of two intersubunit bridges, B2a and B3 [29]. Our data show that the top part of helix 44 is displaced and protrudes from the interface side of the 30S subunit, blocking the final formation of the decoding center and joining of the 50S subunit. These data are in agreement with the results of hydroxyl radical footprinting experiments [12] and cryo-EM reconstruction of the 30S•RbfA complex [9] where it was shown that the upper segment of helix 44 remained undocked.

In previous studies, it has been established that the 30S subunit assembly nucleates at multiple points along the rRNA [30,31,32]. Isolation of similar intermediates from the strains with a knockout of different protein factors led to the firm opinion that the assembly of the 30S subunit proceeds via multiple alternative parallel pathways and many assembly factors could be functionally interchangeable. Analysis of predominant pre-30S intermediates allows us to offer an important update to the models of factor-driven 30S maturation.

**Figure 5:**
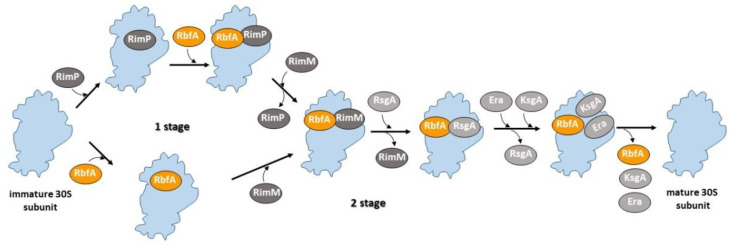
Mechanistic model of RbfA involvement in the 30S subunit assembly. RbfA participates in two distinct assembly stages: early formation of the central pseudoknot including folding of the head, and positioning of helix 44 in the decoding center at a later stage.

It is known that deletion of assembly factor RimP causes a striking defect in central PK stability and a head unanchored to the body and platform [28]. Our results show that RbfA also affects the formation and stabilization of the central PK and folding of the head domain. RbfA might be among the first factors entering the 30S subunit assembly process or may act synergistically with RimP to stabilize the central PK after RimP binding, i.e., alternative pathways are possible when the formation of the central PK precedes folding of the head and vice versa. We assume that it is RimM that enters the scene after the stabilization of the central PK. It was shown that the head domain in the Δ*rimM* pre-30S particle is dramatically rotated and RimM was considered to precede RbfA only because structural data on pre-30S intermediates from double mutant Δ*rbfA*Δ*rsgA* revealed fewer problems with the head domain rotation [24]. The presence of several intermediates lacking a head domain may indicate the opposite order, RimM after RbfA, and the overlapping of the factors’ functions. Previously published models implied that after RimM leaves the scene, factors RsgA, Era, and KsgA come into play together or one by one [24,25,33]. We propose an update to the existing models (Figure 5), assuming that RbfA participates in most of the 30S assembly process, affecting both the early formation of the central pseudoknot including folding of the head, and positioning of helix 44 in the decoding center at a later stage, probably preventing it from docking against the body before the central pseudoknot has formed. The presence of almost mature intermediates (class F) with displaced helix 44 confirms this assumption. Apparently, bound RbfA prevents final docking of helix 44 until the maturation of the 30S subunit is completed, serving as a checkpoint for the particles ready for translation.

## 4. Materials and Methods

### 4.1. E. coli Strains

*E. coliK*-12 derivative MG-1655 (F- lambda- *ilvG*- *rfb*-50 *rph*-1) was used as a wild-type reference [34]. The Δ*rbfA* strain JW3136-1 was BW25113 (F^-^ DE(araD-araB)567 lacZ4787(del)::rrnB-3 LAM^-^ rph-1 DE(rhaD-rhaB)568 hsdR514 ∆*rbfA::kan*) was obtained from the Keio collection, a set of *E. coli* K-12 in-frame, single-gene knockout mutants [14].

### 4.2. Isolation of Wild-Type 30S Ribosomal Subunits and Immature ΔrbfA 30S Particles

30S subunits from the reference strain MG 1655 were obtained by dissociation of 70S ribosomes. Briefly, 70S ribosomes purified according to [15] with minor modifications were dissociated into subunits under low magnesium concentration, fractionated in 15–30% (*w*/*v*) sucrose gradient, and the fractions containing 30S subunits were collected.

Immature Δ*rbfA* 30S particles from the Δ*rbfA* strain unable to associate with 50S subunits were purified by sucrose gradient centrifugation of the total ribosome fraction in regular salt conditions. The small ribosomal subunit fraction was considered to consist mainly of immature particles.

See Supplementary Materials for the detailed description of the procedures.

### 4.3. Two-Dimensional Gel Electrophoresis

To assess the protein content of ribosomal preparations, proteins were extracted by the acetic acid method and analyzed by two-dimensional gel electrophoresis using acidic–acidic system IV [35].

### 4.4. Cryo-Sample Preparation

For cryo-EM analysis, the 30S particles were further purified by gel-filtration with a Superose^®^ 6 Increase 10/300 column (GE Healthcare, Uppsala, Sweden) equilibrated with HAKM7 buffer (50 mM HEPES-KOH, pH 7.5, 70 mM NH_4_Cl, 30 mM KCl, 7 mM MgCl_2_). The 30S-containing fractions were pooled and pelleted by centrifugation in an SW 55 rotor (Beckman Coulter, Brea, CA, USA) at 50,000 rpm, for 3 h. The 30S particles were dissolved in HAKM7 buffer, aliquoted, frozen in liquid nitrogen, and stored at −70 °C. Before the application to an EM grid, wild-type 30S subunits were reactivated by a 30 min incubation at 37 °C at a high Mg^2+^ concentration (HAKM20, 20 mM MgCl_2_) and all the samples were diluted to 0.4 µM of 30S particles in HAKM7.

### 4.5. Cryo-EM Data Collecting

Quantifoil R 2/2 grids coated with an additional 5 nm amorphous carbon film were glow-discharged for 30 s at 15 mA using PELCO easiGlow (Ted Pella, Inc., Redding, CA, USA). Three microliters of the sample were applied onto the grids, blotted for 3 sec at 10 °C and 100% humidity, and plunge-frozen in liquid ethane using Vitrobot Mark IV (Thermo Fisher Scientific, Inc., Hillsboro, OR, USA) [36].

Cryo-EM data were collected using a Cs-corrected Titan Krios (Thermo Fisher) transmission electron microscope, equipped with a Falcon II direct electron detector. Data were acquired with defocus range of −0.6 to −2.0 at a nominal magnification of 75,000×, giving a calibrated pixel size of 0.86 Å/pixel. The micrographs were recorded as movie stacks. The exposure time for each stack was 2 s for wild-type 30S subunits and 1.6 s for Δ*rbfA* 30S subunits, corresponding to a total electron dose of ~80 e^−^/Å^2^ fractionated into 40 frames (~2 e^−^/Å^2^ per single frame) and 32 frames (~2.5 e^−^/Å^2^ per single frame), respectively. In total, 3571 and 3640 movie stacks were collected for wild-type 30S subunits and Δ*rbfA* 30S subunits, respectively.

### 4.6. Cryo-EM Processing

Drift correction using all frames on 5 patches, dose-weighting, per frame local CTF estimation, as well as automated particle picking using a re-trained deep learning-based BoxNet method, were performed with Warp software [37]. Then, 2944 (for wild-type 30S subunits) and 3351 (for Δ*rbfA* 30S subunits) images under a 3.0 Å estimated CTF resolution threshold were used for further data processing, resulting in 489,736 and 514,927 particles, respectively.

All further data processing steps were performed using cryoSPARC v3.1.0/v3.2.0 software [38]. For both datasets, multiple rounds of reference-free 2D classification were performed using 50 classes at each step to minimize the number of false-positive particles in the subset and to remove non-30S subunit particles (50S subunits specifically, which represented less than 3% of particles in the case of Δ*rbfA* 30S subunit preparation). The results of heterogeneous ab initio reconstruction were used as an initial reference. In the case of wild-type 30S subunits, heterogeneous dataset refinement with 2 classes was performed to separate particles with a preferred orientation. Thus, for consensus refinement, 169,371 (for wild-type 30S subunits) and 352,365 (for Δ*rbfA* 30S subunits) particles were used, resulting in the maps of 3.2 Å and 2.7 Å resolution, respectively, estimated using an FSC = 0.143 gold-standard threshold. To analyze the structural heterogeneity of the sample, 3D variability analysis [39] with three main components was performed for both datasets. For Δ*rbfA* 30S subunits, several 3D classification strategies were tried. As a result, we settled on the clustering with 4 clusters in the reaction coordinate space using a Gaussian mixture model and then used these maps and two additional consensus maps as initial models for heterogeneous refinement. For each of the resulting classes, non-uniform refinement [40] was conducted. For wild-type 30S subunits and the particles of the most populated class F, local refinement was performed separately for the head and the body parts after CTF refinement and signal subtraction from the corresponding regions. For WT 30S subunits, a final local resolution was estimated as 3.1 Å for the body and 4.6 Å for the head, and for the class F intermediate—2.7 Å and 2.6 Å, respectively (Supplementary Materials Figures S2 and S3). All maps were locally filtered based on the local resolution estimation procedure implemented in the cryoSPARC software package.

### 4.7. Model Building

For model building, the 30S subunit (chain A) was extracted from a *E. coli* vacant 70S ribosome crystal structure model (PDB ID 4V4Q). Head and body domains of the 30S subunit were fitted into cryo-EM maps as a rigid body using UCSF Chimera software [41]. The initial course fitting of flexible elements into the density was performed using the Namdinator web service (https://namdinator.au.dk (accessed on 8 March 2021)). The default parameters were used for flexible fitting. Models and maps were visually inspected in the Coot program [42]. The final models were built after several rounds of real space refinement in the Phenix program suite [43] and manual model building in Coot. For model validation, we used MolProbity from Phenix. Data and refinement statistics are summarized in Supplementary Materials Table S1.

## Data Availability

The cryo-EM maps have been deposited in the EMDataBank (EMDB codes 12855 for class A; 12858 for class D; 12854 for class E; 12856 for class F; and 12857 for wild-type 30S subunit). The coordinates for the atomic models built for the wild-type 30S subunit and the Δ*rbfA* 30S particles of classes D and F have been deposited in the Protein Data Bank (PDB codes ID 7OE0 for class F; ID 7OE1 for wild-type 30S subunit).

## Supplementary Materials

The following are available online at https://www.mdpi.com/article/10.3390/ijms22116140/s1, Reference [44] is cited in the supplementary materials.
