# Supplementary material for: RbfA Is Involved in Two Important Stages of 30S Subunit Assembly: Formation of the Central Pseudoknot and Docking of Helix 44 to the Decoding Center
Source: Int J Mol Sci. 2021 Jun 7;22(11):6140. doi: 10.3390/ijms22116140 (PMC8201178; doi:10.3390/ijms22116140)
Supplement: Supplementary file 1 [file ijms-22-06140-s001.zip › ijms-1225921-supplementary.pdf]

Supplementary Materials

RbfA is involved in two important stages of 30S subunit assembly: formation of the central pseudoknot and docking of helix 44 in the decoding center

Elena M. Maksimova, Alexey P. Korepanov, Olesya V. Kravchenko, Timur N. Baymukhametov, Alexander G. Myasnikov, Konstantin S. Vassilenko, Zhanna A. Afonina and Elena A. Stolboushkina

This file includes:

I. Supplementary Figure S1, S2, S3 with legend

II. Supplementary Table S2

III. Supplementary Materials and Methods

I. SUPPLEMENTARY FIGURE

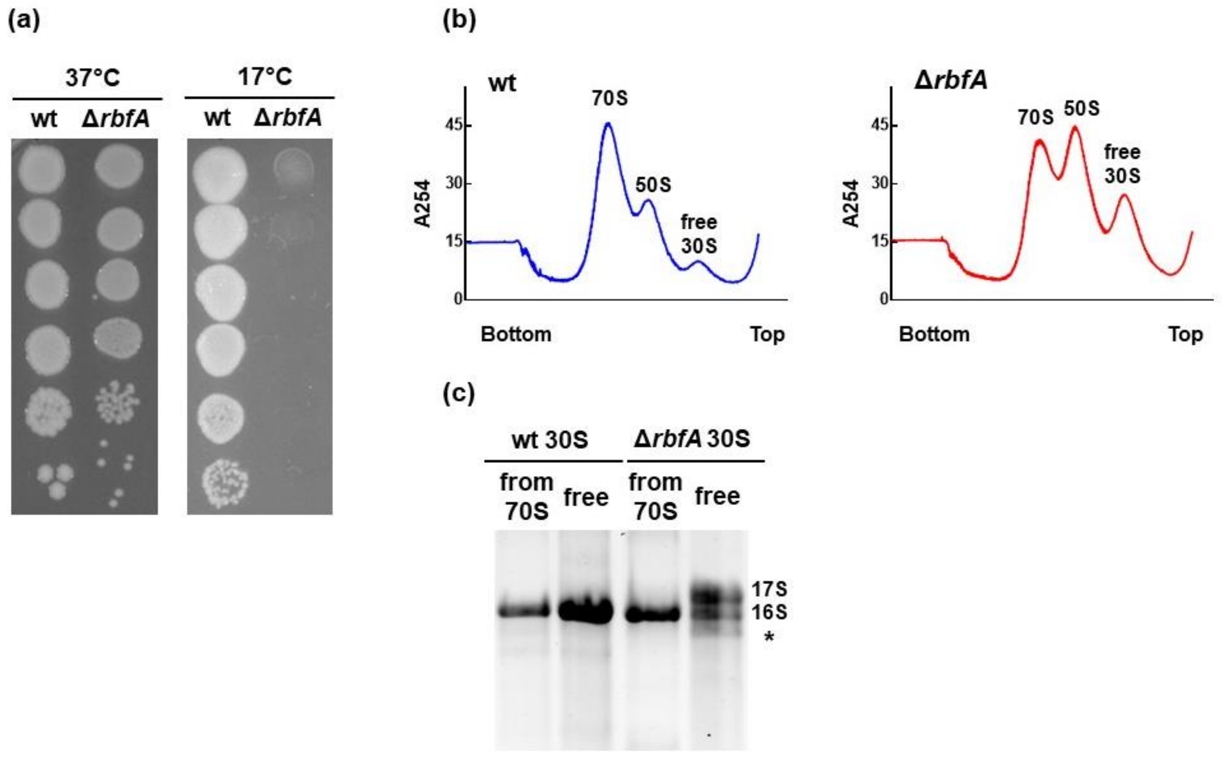

Supplementary Figure S1. Comparison of wild-type (wt) and Δ*rbfA* strains of *E. coli*. **(a)** Bacterial growth on LB-agar plates at 37 and 17°C (consecutive dilutions in 1:10 steps). **(b)** Sucrose density gradient fractionation of the lysates of wild-type and Δ*rbfA* cells collected at exponential growth phase. **(c)** Electrophoretic analysis of rRNA from small ribosomal subunits purified from 30S and 70S fractions of WT and ΔrbfA cell lysate preparations. Asterisk indicates the result of degradation caused by unformed central pseudoknot [27].

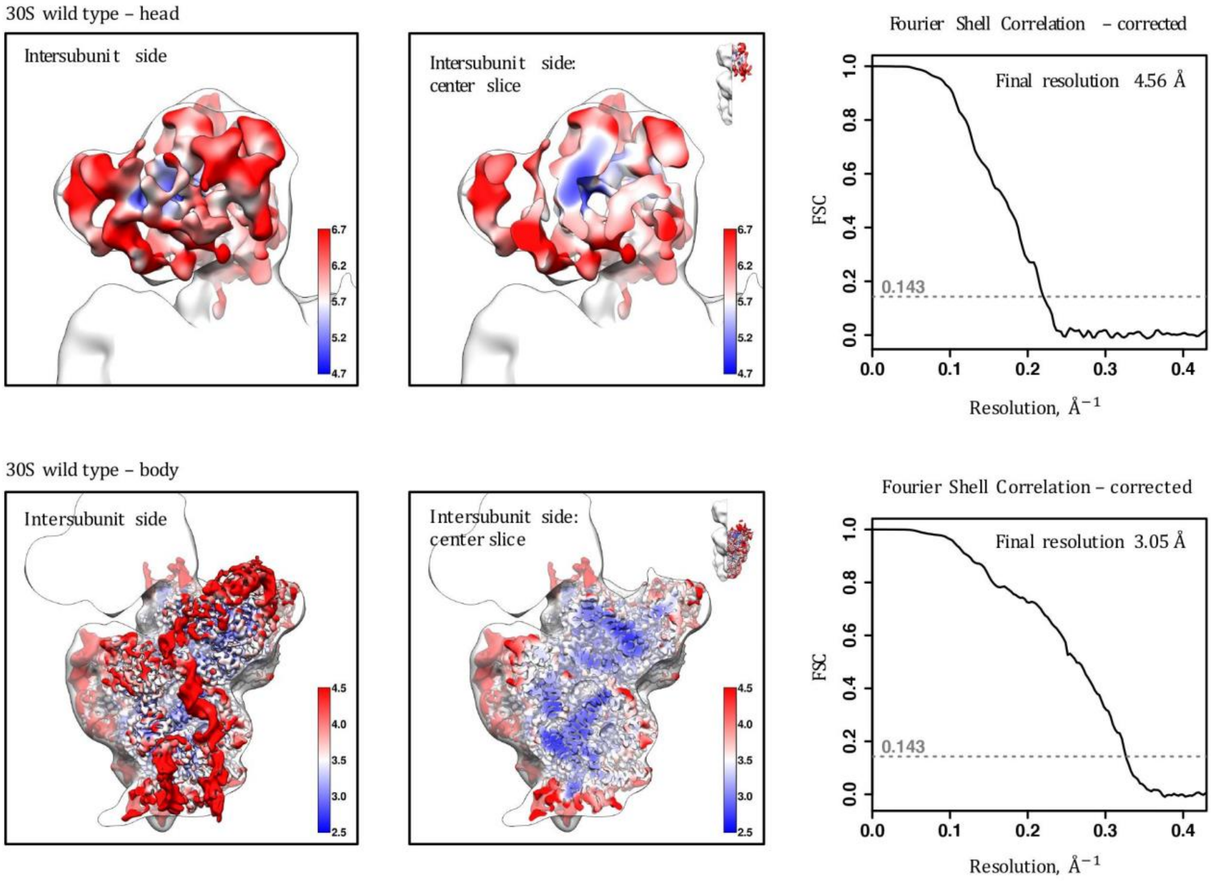

Supplementary Figure S2. Cryo-EM maps of locally refined WT 30S head (top) and body (bottom). Maps are colored (color key is given in angstroms) and filtered according to local resolution, and corresponding "gold standard" FSC=0.143 curves for corrected refinement masks are shown. Outlined transparent surface of the corresponding consensus map are shown for demonstration. Focused local refinement improved the resolution of the body from 3.2 Å to 3.1 Å, resolution of the 30S head was improved from approx. 10 Å to 4.6 Å.

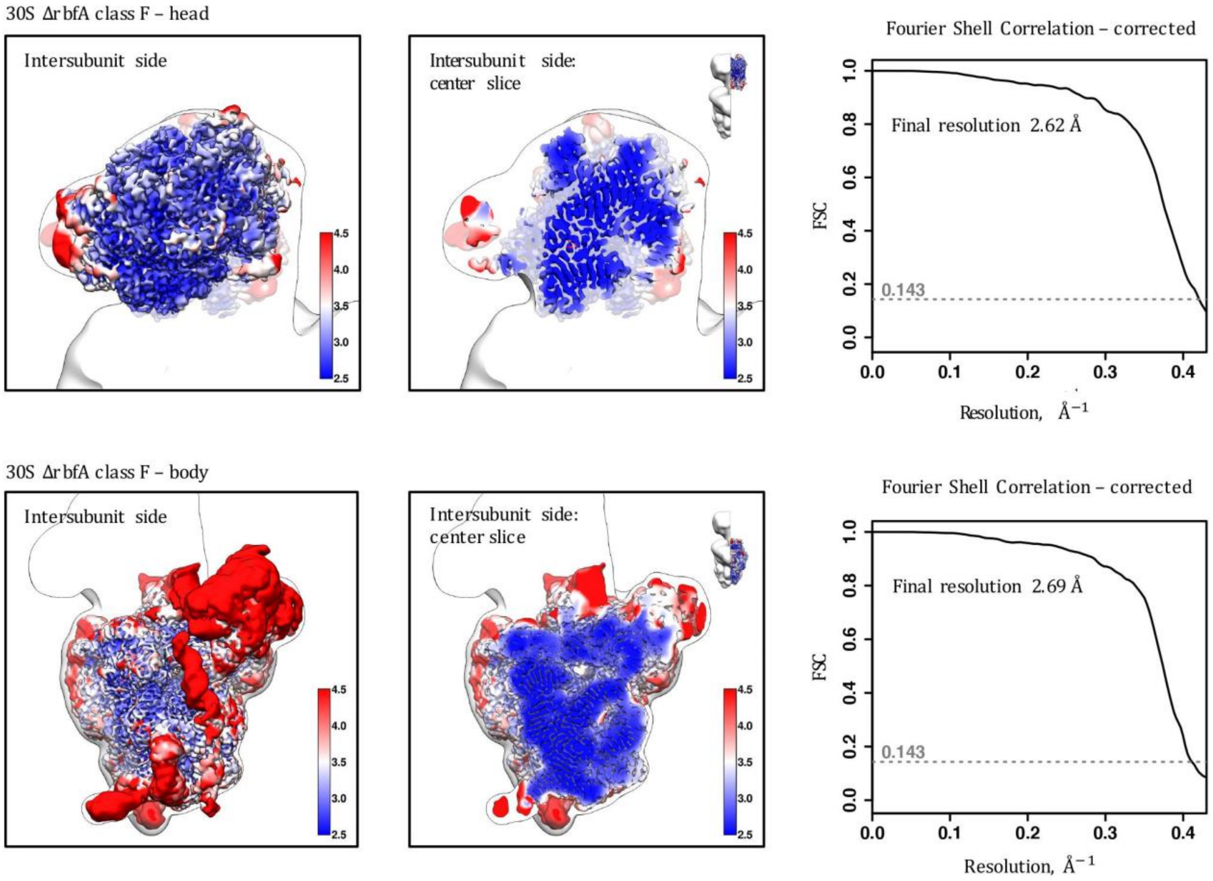

Supplementary Figure S3. Cryo-EM maps of locally refined Δ*rbf*A 30S head (top) and body (bottom). See legend to Figure S2. Resolution of the body changed insignificantly, resolution of the head was improved from approx. 10 Å to 2.6 Å.

II. SUPPLEMENTARY TABLE

Table S1. Cryo-EM data collection, refinement and validation statistics.

	30S WT	pre-30S Δ*rbfA*	
Data collection and processing			
Nominal magnification	75,000x	75,000x	
Voltage (kV)	300	300	
Electron exposure (e-/Å^2)	2	1.6	
Nominal defocus range (um)	-2.0 to -0.7	-2.0 to -0.7	
Pixel size (Å)	0.86	0.86	
Initial particle images	489736	514927	
Final particle images	169371	106319 (class D), 138247 (class F)	
Symmetry imposed	C1	C1	
FSC threshold	0.143	0.143	
Map resolution (Å)	3.05 (body), 3.54 (head)	2.76 (class D), 2,69 (class F)	
Refinement			
	30S WT	pre-30S Δ*rbfA* class D	pre-30S Δ*rbfA* class F
Initial model used	4V4Q	4V4Q	4V4Q
Model resolution (Å)	3.2	2.8	2.8
Model composition			
Non-hydrogen atoms	51092	29257	50300
Protein residues	2320	1037	2208
nucleotides	1530	963	1510
water	0	374	530
B-factors (Å^2)			
protein	58.73	17.07	18.95
RNA	72.21	28.32	34.60
r.m.s. deviation			
Bond length (Å)	0.08	0.014	0.014
Bond angles (°)	0.923	1.076	1.064

validation			
MolProbity score	2.65	2.42	2.43
Clashscore	32.17	23.53	24.00
Poor rotamers (%)	0	0.23	0
Ramachandran plot			
Favored (%)	84.86	89.77	89.84
Allowed (%)	15.14	10.23	10.16
Disallowed (%)	0	0	0

III. SUPPLEMENTARY MATERIALS AND METHODS

S1. Isolation of wild-type 30S ribosomal subunits and immature 30S ΔrbfA particles

Overnight cultures were grown from isolated fresh colonies and used to inoculate 10 liters of the LB medium (Difco) at 1:200 dilution. Cultures were grown at 37 °C with vigorous shaking to OD_{600} ~ 0.6 for the reference strain MG 1655 and to OD600 ~ 0.2 for *ΔrbfA* strain, chilled in an ice-water bath, and all subsequent procedures were carried out at 4 °C. Cells were harvested at 5000 g for 25 min, washed in buffer A (20 mM Tris-HCl, pH 7.5, 100 mM NH_4Cl, 10 mM $MgCl_2$), re-pelleted and frozen at -70 °C.

Wild-type cell pellet was resuspended in buffer B (20 mM Tris-HCl, pH 7.5, 100 mM NH_4Cl, 10.5 mM $MgCl_2$, 0.5 mM EDTA, 3 mM 2-mercaptoethanol) and ground with alumina powder (2 grams of Al_2O_3 per 1 gram of cell pellet). Debris was removed by double centrifugation in the 70 Ti rotor (Beckman Coulter): first, at 30 000 rpm for 30 min; second, at 17 600 rpm for 35 min. The supernatant was layered on top of 9 ml of 1.1 M sucrose cushion in buffer C (20 mM Tris-HCl, pH 7.5, 500 mM NH_4Cl, 10.5 mM $MgCl_2$, 0.5 mM EDTA, 3 mM 2-mercaptoethanol) in 26.3 ml polycarbonate flasks and centrifuged in the 70 Ti rotor at 40 000 rpm for 14 h. Ribosome pellet was dissolved in buffer C and the ribosome suspension was layered over the 1.3 ml of the sucrose cushion and centrifuged in the 70 Ti rotor at 65 000 rpm for 4 h 40 min. Pelleted ribosomes were dissolved in buffer C and loaded on top of 1.6 ml sucrose cushion and centrifuged in the SW 28 rotor (Beckman Coulter) at 25 000 rpm for 16 h. Ribosomes were dissolved in buffer C, layered over 15-30% (w/v) sucrose density gradient in buffer D (20 mM Tris-HCl, pH 7.5, 60 mM NH4Cl, 5 mM MgCl2, 3 mM 2-mercaptoethanol) and centrifuged in the SW28 rotor at 24 000 rpm for 12 h. The gradients were fractionated using BT100-1F peristaltic pump (LongerPump, China) and Model 2110 fraction collector (Bio-Rad Laboratories). Ribosome profiles were monitored by UV absorbance at 260 nm. Fractions,

containing 70S ribosome were pooled and centrifuged in the 70 Ti rotor at 50 000 rpm for 14 h. The ribosome pellet was dissolved in the buffer $TAKM_7$ (50 mM Tris-HCl, pH 7.5, 70 mM NH_4Cl, 30 mM KCl, 7 mM $MgCl_2$). To obtain 30S subunits, 70S ribosomes were dissociated under low magnesium concentration conditions as follows: ribosome suspension was layered over 15-30% (w/v) sucrose gradient in buffer E (20 mM Tris-HCl, pH 7.5, 60 mM NH_4Cl, 1 mM $MgCl_2$, 3 mM 2-mercaptoethanol) and centrifuged in the SW 28 rotor at 24 000 rpm for 12 h. Fractions containing 30S subunits were pooled and centrifuged in the 70 Ti rotor at 50 000 rpm for 16 h. The 30S subunits were dissolved in the $TAKM_7$, aliquoted, frozen in liquid nitrogen and stored at -70 °C. The sample concentration was determined by measuring UV absorbance at 260 nm.

Immature 30S particles from *ΔrbfA* cells were purified identically up to the 70S ribosome separation from non-associated subunits. At this step, fractions corresponding to free 30S subunits were polled and centrifuged in the 70 Ti rotor at 50 000 rpm for 16 h. The subunits were dissolved in the $TAKM_7$, aliquoted, frozen in liquid nitrogen and stored at -70°C. Concentration of ribosomal material was determined by monitoring UV absorbance at 260 nm.

S2. Gel electrophoresis assay

rRNA from free immature 30S subunits or mature 30S subunits from 70S ribosomes purified by sucrose gradient centrifugation [44] was extracted by vortexing with equal volume of phenol and subsequent centrifugation at 10 000 g, 10 min. Part of water phase was mixed with loading dye solution (1x TBE buffer, 10% sucrose, 0.36% bromphenol blue and 0.36% xylene cyanol) and loaded on the gel composed of 0.7% agarose and 0.9% Synergel in 0.5x TBE buffer. rRNA was separated at 80V, 3 h, and stained in 1 µg/ml ethidium bromide.
